# Supplementary material for: Learning curve and surgical outcome of robotic assisted colorectal surgery with ERAS program
Source: Sci Rep. 2022 Nov 29;12:20566. doi: 10.1038/s41598-022-24665-w (PMC9709162; doi:10.1038/s41598-022-24665-w)
Supplement: Supplementary file 1 — Supplementary Information 1. [file 41598_2022_24665_MOESM1_ESM.zip › LSC Raw data-Ñ[▒K/147/H_ñw╝╨░O▒Kñσ.pdf]

正式摘要(電子簽章：00000023408l443)

W92 / W92 ☐ 英文標題(注意:無英文姓名-請補病人基本檔!) ☐【讀取】憑證

臺中榮民總醫院
出院病歷摘要

轉入號： 轉出號：

[redacted]

(8)流水編號	
(9)入院日期	110/12/29 (2021) 直腸外科 W92 - 37
(10)轉科日期	
(11)出院日期	111/01/08 (2022) 住院天數計 10 日

診斷

- 入院:sigmoid colon cancer
- 出院:sigmoid colon cancer

主訴

- diarrhea and bloody stool for 2 months

病史

- This is a 66 years old female patient with history of

  1. Thyroid cancer s/p total thyroidectomy over 30 years ago

  2. s/p Tubectomy(?) over30 years ago

  According to the patient, she suffered from diarrhea and bloody stool for 2 months. She visit LinShin H. first, where colonoscopy with biopsy proved S-colon adenocarcinoma. Abdominal CT was done there, revealed S-colon cancer with suspected R't adnexa invasion. Suspected L't adnexa lesion was also seen. PET was then arranged but showed no other specific findings except S-colon cancer.

  Under the impression of S-colon cancer, she was admitted to our ward for operation.

體檢發現

- ADMITTED FOR TPN AND OP NEXT WEEK, CONSULT GYN, TRY LAP
  WELL EXPLAIN THE RISK OF CONVERSION
  - General appearance :
    - easy looking , conscious clear , without cardiopulmonary distress
    - Vital signs自動帶入date 體溫(BT) 脈搏(P) 呼吸(R) 血壓(BP) 身高(BH) 體重(BW) BMI
      20211229 36.4C 83/min 16/min 135/77mmHg 159.9cm 66.5kg 26.0
  - HEENT :
    - Eyes: no anemic conjunctiva, no icteric sclera, normal EOM
    - ENT: no otorrhea, no rhinorrhea, no erythematous mucosa, no injected tonsil, no deformity
  - Neck: supple, freely movable, trachea midline,no deformity, no engorged jugular vein , no palpable thyroid or lymph node enlargement
  - Chest:
    - Inspection: Grossly normal, symmetric and fully expansion, no accessory muscle used, no operation scar, no spider angiomata, no gynecomastia
    - Palpation: no palpable mass or nodule, no subcutaneous emphysema, no tenderness
    - Ausculation: clear breathing sound
  - Heart:
    - Percussion: normal heart size
    - Ausculation: regular heart beat, normal S1, S2, no gallop, no murmur

-
  - Abdomen:
    - Inspection: in
      - flat contour, symetrically, no scar, no stria, no dilated veins, no rashes
    - Ausculation: normoactive bowel sound, no abdominal bruits
    - palpation: soft, no muscle guarding or rigidity, no local tenderness, no rebounding pain, no palpable mass, no palpable pulsation
    - Percussion: no shifting dulllness, no hepatomegaly, no splenomagaly
  -

Peripheral pulsation :	CA	BA	RA	FA	PA	PTA	DPA
Right side :	++	++	++	++	++	++	++/+++
Left side :	++	++	++	++	++	++	++/+++

  - Genito-anus: no tenderness or palpable mass lesion or rectal shelf, normal external genitalia, normal anal tone, brown stool

CRS CONDITION

-
  - Lung

手術日期及方法

- 手術日期及方法20220103 Laparoscopic low anterior resection of rectum

住院治療經過

- This is a 66 years old female patient with history of
  1. Thyroid cancer s/p total thyroidectomy over 30 years ago
  2. s/p Tubectomy(?) over30 years ago keep monitor vital sign and low residual diet for pending operation on 12/29-1/2 colon prepare was done on 1/2, the Laparoscopic low anterior resection was done on 1/3. After the operation,we keep monitor patient's vital sign, pain score and wound condition. lab data was followed up on 1/5 WBC:10940 HGB:10.5 HCT:31.2 PLATE:232 NEUT:88.5 LYM:8.0 MONO:2.5 EOSINO:0.8 BASO:0.2 ALB:2.9 BILT:0.73 BILD:0.20 ALKP:41 GPT:7 NA:138 K:3.5 CL:102 CA: 5.4-->6.0 BUN:5 CREAT:0.60 CRP:8.437 GFR:106.31 the condition of hypoclacemia had improved but not enough so we closely monitor her serum electroltyes level and keep the calcium gluconate QD. Patient looked fair and the condition is stable after surgery so she was allowed to discharge on 1/8 and follow up at OPD.

合併症

一般檢查

特殊檢查

病理報告

其它

出院時情況

- 3.改門診治療

出院指示

-

藥名	劑量	劑量單位	用法	頻次	數量	數量單位	現況	天數	單位
120mL (C) B.M. sol	5	ML	PO	TID	1	ML	TH	5	天
0.05mg Eltroxin tab	3	TAB	PO	QDAC	15	TAB	TH	5	天
Ultracet tab	1	TAB	PO	Q6H	20	TAB	TH	5	天
(□)Takepron OD tab 30mg	1	TAB	PO	QDAC	5	TAB	TH	5	天

出院計劃

主治醫師 2062F 蔣鋒帆

正式摘要(電子簽章：**000000234081443)**

		W92 / W92				

doctorRank:B
